# Low Gain Values of the Vestibulo-Ocular Reflex Can Optimize Retinal Image Slip

**DOI:** 10.3389/fneur.2022.897293

**Published:** 2022-07-12

**Authors:** Stefan Glasauer, Hans Straka

**Affiliations:** ^1^Computational Neuroscience, Institute of Medical Technology, Brandenburg University of Technology Cottbus-Senftenberg, Cottbus, Germany; ^2^Brandenburg Faculty for Health Sciences, Brandenburg University of Technology Cottbus-Senftenberg, Cottbus, Germany; ^3^Faculty of Biology, Ludwig-Maximilians-University Munich, Planegg, Germany

**Keywords:** semicircular canal, eye movement, extraocular motoneuron, optokinetic reflex, *Xenopus laevis*, sensorimotor noise

## Abstract

The angular vestibulo-ocular reflex (aVOR) stabilizes retinal images by counter-rotating the eyes during head rotations. Perfect compensatory movements would thus rotate the eyes exactly opposite to the head, that is, eyes vs. head would exhibit a unity gain. However, in many species, but also in elderly humans or patients with a history of vestibular damage, the aVOR is far from compensatory with gains that are in part considerably lower than unity. The reason for this apparent suboptimality is unknown. Here, we propose that low VOR gain values reflect an optimal adaptation to sensory and motor signal variability. According to this hypothesis, gaze stabilization mechanisms that aim at minimizing the overall retinal image slip must consider the effects of (1) sensory and motor noise and (2) dynamic constraints of peripheral and central nervous processing. We demonstrate that a computational model for optimizing retinal image slip in the presence of such constraints of signal processing in fact predicts gain values smaller than unity. We further show specifically for tadpoles of the clawed toad, *Xenopus laevis* with particularly low gain values that previously reported VOR gains quantitatively correspond to the observed variability of eye movements and thus constitute an optimal adaptation mechanism. We thus hypothesize that lower VOR gain values in elderly human subjects or recovered patients with a history of vestibular damage may be the sign of an optimization given higher noise levels rather than a direct consequence of the damage, such as an inability of executing fast compensatory eye movements.

## Introduction

**Figure 1:**
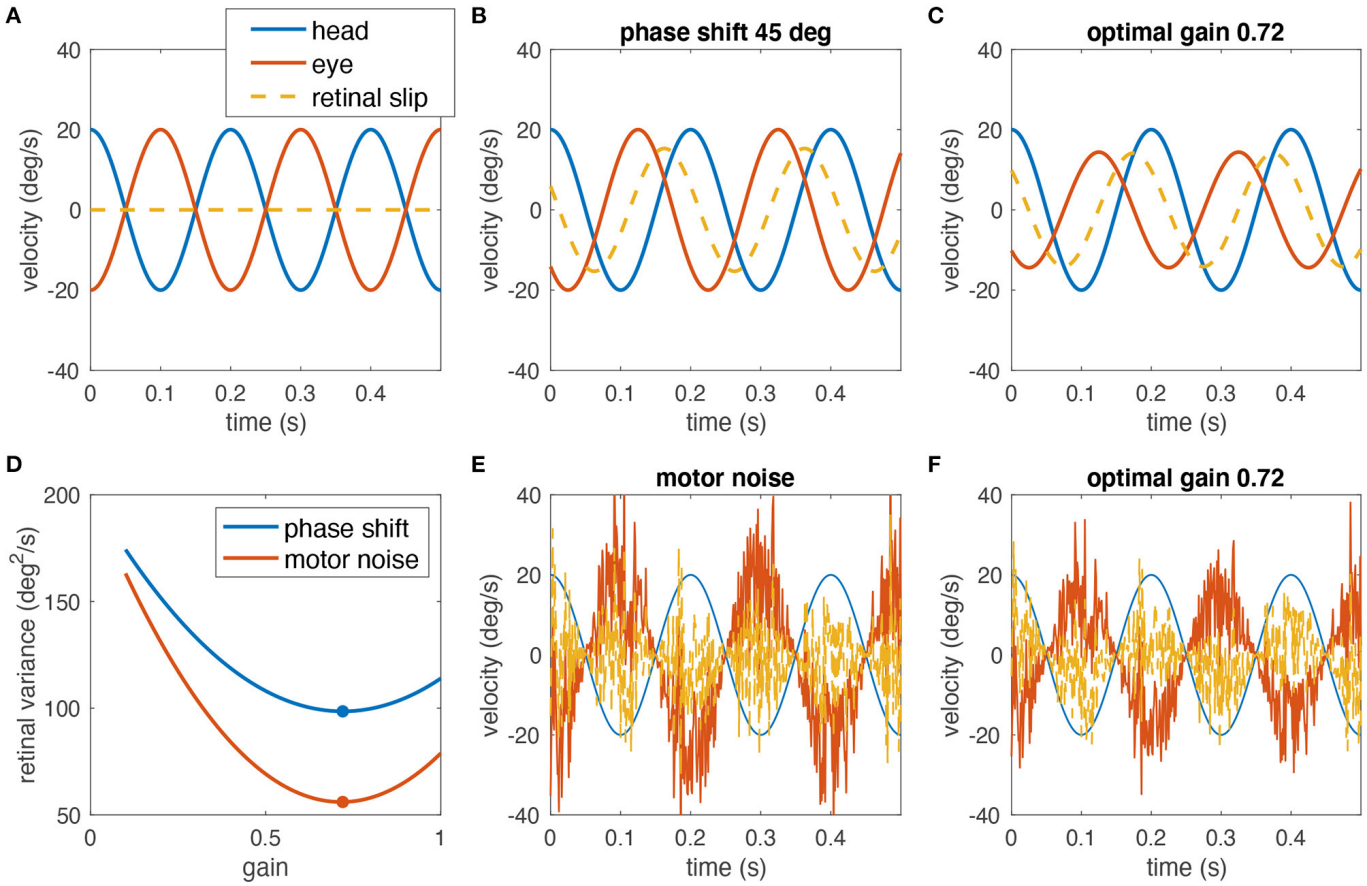
Simulated example of why decreasing the gain of the VOR can help minimizing the retinal image slip. **(A)** A perfect compensatory VOR (gain = 1, no phase shift, no noise) results in zero retinal image slip (eye velocity in red, head velocity in blue, retinal image slip in dashed yellow). **(B)** In the presence of a 45° phase shift between eye and head rotation, a strong retinal image slip is present despite a gain of 1. **(C)** Reducing the VOR gain to 0.72 minimizes the overall retinal image slip. **(D)** Variance of the retinal slip plotted with respect to the VOR gain for phase shifts as in **(B,C)** (blue), and noise as in **(E,F)** (red). The plots have been obtained from numerical simulations. The optimal gain in both cases is ~0.72 (red and blue dots). **(E)** Signal-dependent motor noise, proportional to eye velocity introduces a strong retinal image slip even if the gain = 1. **(F)** Reduction of the VOR gain also reduces the motor noise and thus the overall retinal image slip; retinal image slip plotted with respect to the VOR gain for this example is shown in **(D)** (red curve).

The angular vestibulo-ocular reflex (aVOR) is driven by semicircular canal afferent signals and becomes effective by counter-rotating the eyes during head movements, thereby stabilizing retinal images. A perfect compensatory stabilization would thus require that the eye movements have the same velocity as the head movement, only in the opposite direction, that is, eye vs. head motion should exhibit a VOR gain of −1 (gain: ratio of eye angular velocity to head angular velocity) (Figure 1A). Indeed, a VOR gain of (in absolute value) unity or very close to it can usually be found in healthy young humans. However, healthy elderly subjects (1) or patients with a clinical history of vestibular damage or dysfunction (2) can exhibit lower gain values, even though an age-related decline of the VOR gain in humans is relatively moderate or may even be absent (3, 4). In many non-human vertebrate species, the VOR is far from compensatory with gains clearly lower than unity. For example, in rabbits, rotation at a frequency of 0.76 Hz is accompanied by eye movement gains of 0.57 (5), and in wildtype mice, during sinusoidal head rotation at a frequency of 0.8 Hz the VOR gain in darkness ranges from ~0.4 (6) to ~0.8 (7) largely depending on the eye motion recording technique. However, the reason for this is not that sufficiently fast eye movements cannot be generated: the mouse VOR gain in light at 0.8 Hz is ~0.8 (6) to >0.9 (7), demonstrating that adequate gaze stabilization is not limited by the properties of the motor system. To the best of our knowledge, the reason for the apparent suboptimality of the VOR gain is so far unclear.

To explain this conundrum, we hypothesize that gain values smaller than unity can in fact be the optimal solution for gaze stabilization for two reasons. First, neural processing subserving the VOR includes the high-pass dynamics of the semicircular canals (partly compensated by the velocity storage mechanism) and elements such as the neural integrator that both can introduce phase shifts, which in turn increase retinal image slip at some frequencies due to the delay between head and eye movements (for an example, see Figures 1B–D). Consequently, if retinal image slip, due to the phase lag, must be avoided, a small gain may be desirable. However, in rabbits, the phase shift of the VOR at rotations with a frequency of 0.76 Hz was only 2.5°, by far too small to correspond to a gain of 0.57 (5). Therefore, a second aspect must be considered: signal-dependent sensory and motor noise (8) induces retinal image slip, and increases for faster and larger movements, which could outweigh the benefit of an appropriate compensatory gain value (example Figures 1D–F). Again, a smaller VOR gain would be more beneficial, because it would reduce the motor noise and consequently also the retinal image slip. This suggests that small VOR gain values are not necessarily the consequence of a general incapacity of a particular species to exert sufficiently large or fast eye movements but rather a strategy to minimize retinal image slip.

Here we tested this hypothesis by investigating the aVOR in *Xenopus laevis* tadpoles, which show a particularly small VOR gain at mid-larval stages despite being able to make fast and large eye movements (9–11). While the phase of the aVOR oscillations can be computed from experimentally measured eye movements, neither sensory nor motor noise is directly accessible from eye movement data. If our hypothesis is correct, the variability of eye movements cannot be taken as proxy for sensorimotor noise, because the observed variability would be a consequence of optimization instead of directly reflecting internal noise from sensors or muscles. Therefore, a different approach was necessary to test the hypothesis. Accordingly, we modeled the dynamics of individual VOR responses to infer the internal noise level that corresponds to experimentally observed gain values assuming an optimal state. Using this model and applying sensory and motor noise corresponding to the gain, we were able to predict the variability of the eye position. Finally, these model predictions were then compared with the experimentally observed variability of the actual VOR-related eye movements in *Xenopus* tadpoles. If our hypothesis is true, predicted and observed variability should be quantitatively similar.

## Methods

### Animal Experiments and Data Analysis

For this work we used eye movement data captured from six *Xenopus laevis* tadpoles, which had been published previously (11). The experimental protocol, stimulation and recording paradigms have been reported in full detail in the respective publication (11). All experiments were conducted *in vitro* on semi-intact preparations of *Xenopus laevis* tadpoles at developmental stages 53–54 (12) and complied with the “Principles of animal care,” publication No. 86-23, revised 1985 of the National Institute of Health. All experiments were carried out in accordance with the ARRIVE guidelines and regulations. Permission for these experiments was granted by the ethics committee for animal experimentation of the legally responsible governmental institution (Regierung von Oberbayern) under the license code 55.2-1-54-2532.3-59-12. In addition, all experiments were performed in accordance with the relevant guidelines and regulations of the Ludwig-Maximilians-University Munich. Larvae of either sex were obtained from the in-house animal breeding facility at the Biocenter-Martinsried of the Ludwig-Maximilians-University Munich.

In brief, eye movements of *Xenopus* tadpoles were captured non-invasively during passive sinusoidal head rotations in darkness around an earth-vertical axis at 4 different stimulus frequencies (0.1, 0.2, 0.5, 1 Hz) with a peak velocity of ±30°/s as described previously (11). Two of the animals were not tested at 0.1 Hz. The recording duration varied between 35 s and 163 s depending on animal and stimulus frequency (mean number of stimulus cycles for each frequency: 0.1 Hz: 13.0; 0.2 Hz: 15.7; 0.5 Hz: 30.0; 1 Hz: 56.1). Eye movements were captured from above with a video camera (Grasshopper color; Point Gray Research) at a frame rate of 50 Hz (using FlyCap2) and analyzed offline using a custom-written video-processing algorithm (Matlab, The Mathworks) to compute eye position [for details, see (13)]. The raw eye position data was up-sampled to 1,000 Hz, slow drift was removed by piecewise cubic interpolation (Matlab function *makima*) and outliers (e.g., occasional fast phases) were discarded. The resulting eye position trace was averaged over all stimulus cycles and fitted to the averaged stimulus trace to determine gain and phase of the eye movements for each stimulus frequency.

In addition, the average variability of the eye position was computed from the averaged traces for comparison with model predictions (see also below). For ***m*** stimulus cycle repetitions with ***n*** time points in each cycle, first the standard deviation **σ**_***i***_ of the eye position at time point ***i*** over all ***m*** repetitions was computed, then the average $\bar{\sigma }$ over all ***n*** time points was used as variability measure:


$$\sigma_{i}\;=\;\frac{1}{m-1}\sqrt{\sum_{j=1}^{m}{\left(p_{j,i}-{\bar{p}}_{i}\right)}^{2}};\;\bar{\sigma }=\;\frac{1}{n}\sum_{i=1}^{n}\sigma_{i}$$

with ***p***_***j*,*i***_ being the eye position value at time point ***i*** and cycle ***j***, and ${\bar{p}}_{i}$ the eye position at time point ***i*** averaged over all cycle repetitions. Thus, a possible dependency of variability across the cycle will not be visible in the variability measure $\bar{\sigma }$.

### Mathematical Modeling

The model was constructed following classical aVOR models [see (14) for review] with semicircular canal signals as input (modeled as high-pass filter with a time constant of 5 s), the eye plant as final motor output [dominant time constant of 0.33 s and two non-dominant time constants, which can be interpreted as muscle activation, see (11)], and a brainstem processing algorithm by a leaky velocity-to-position integrator and a direct pathway that can be conceived as approximate inverse model of the eye plant. The integrator time constant and the two non-dominant eye plant time constants were fitted as free parameters by minimizing the phase difference between data and model. The overall model gain factor, an additional parameter, which has no influence on the phase of the response, was subsequently fitted to the average gain curve. The model fitting was performed for each animal separately.

The full VOR model, which describes the processing from angular head velocity **ω** to eye position ***p*** can be written in Laplace notation as


(1) $$p\left(s\right)\;=\;-g\;\cdot \;s\cdot \;F_{SC}\left(s\right)\;\cdot \;F_{BS}\left(s\right)\;\cdot \;F_{EYE}\left(s\right)\;\cdot \;\omega (s)$$

with the free gain factor ***g***, semicircular canal highpass ***F***_***SC***_(***s***), brainstem processing ***F***_***BS***_(***s***), and eye plant ***F***_***EYE***_(***s***). The respective model components are:


(2) $$\begin{array}{rcl} F_{SC}\left(s\right) & = & \frac{\tau_{SC}s}{\tau_{SC}s+1} \end{array}$$

(3) $$\begin{array}{rcl} F_{BS}\left(s\right) & = & \tau_{E}+\frac{\tau_{I}-\tau_{E}}{\tau_{I}s+1} \end{array}$$

(4) $$\begin{array}{rcl} F_{EYE}\left(s\right) & = & \frac{1}{\tau_{E}s+1}\cdot \frac{1}{\tau_{M1}s+1}\cdot \frac{1}{\tau_{M2}s+1} \end{array}$$

with the semicircular canal time constant **τ**_***SC***_ fixed to 5 s, leaky integrator time constant **τ**_***I***_ (free parameter), dominant eye plant time constant **τ**_***E***_ fixed to 0.33 s (11), and muscle activation time constants **τ**_***M*1**_ and **τ**_***M*2**_.

Using the fitted model, the tadpole aVOR in response to sinusoidal head rotation at a single frequency (0.5 Hz) was simulated while being contaminated with sensory and motor noise. Both noise sources were modeled as signal-dependent noise (8), that is, the noise amplitude increased proportionally with increasing signal strength. For the sensor noise, the relevant signal was head velocity, for the motor noise it was the motor command signal, that is, the output of the extraocular motoneurons (output of brainstem processing, Equation 3). In the time domain, the noisy motor command ***m***(***t***_***i***_) at time step ***t***_***i***_ can thus be written as


(5) $$\begin{array}{r} m\left(t_{i}\right)\;=\;b\left(t_{i}\right)+\varepsilon_{m}\left(t_{i}\right) \end{array}$$

with ***b***(***t***_***i***_) being the output of the brainstem processing (Equation 3, sum of direct pathway and integrator) and **ε**_***m***_(***t***_***i***_) a normally distributed random number with zero mean and standard deviation ***k***_***m***_ **· *b***(***t***_***i***_). The proportionality constant, or noise factor ***k***_***m***_ thus describes the linear relationship between the standard deviation of motor noise and the motor command. Subsequently ***m***(***t***_***i***_) serves as input to Equation 4 to yield the eye position. Similarly, sensor noise was modeled as **ω**_***n***_(***t***_***i***_) **=** **ω**(***t***_***i***_)**+ε**_***s***_(***t***_***i***_) with **ε**_***s***_(***t***_***i***_) as normally distributed random number with zero mean and standard deviation ***k***_***s***_ **· ω**(***t***_***i***_) and **ω**(***t***_***i***_) as instantaneous head velocity at time step ***t***_***i***_. The noisy head velocity **ω**_***n***_(***t***_***i***_) serves as input to Equations 1 or 2.

From the simulated response, the variance of the retinal image slip was computed. The optimal gain factor that minimizes the variance of retinal image slip was determined by varying the gain factor. Sensory and motor noise were assumed to have equal noise factors, i.e., ***k*** **=** ***k***_***m***_ **=** ***k***_***s***_. The noise factor ***k*** was varied to find noise levels for which the optimal gain factor corresponds to the one that was found in the actual experiments.

As possible alternatives, we also simulated the model (1) with signal-independent (constant) additive sensor noise, (2) with only signal-dependent sensor noise, and (3) with only signal dependent motor noise. The fourth possibility, constant motor noise only, did not yield optimal gain values comparable to the ones found in tadpoles, because constant motor noise cannot be suppressed by small VOR gains.

The model was implemented in Matlab (The Mathworks) as Laplace transfer function using the Signal Processing Toolbox. For each frequency and amplitude, the amplitude and phase of the model response was derived by using the Matlab function *freqresp*. We first fitted the phase of the model response to the experimental data by using the Matlab function *fmincon* with lower and upper limits and the three time constants **τ**_***M*1**_, **τ**_***M*2**_, and **τ**_***I***_ as free parameters. Subsequently, the free overall gain factor ***g*** was fitted to the experimentally determined VOR gain values. For the model simulations, including sensor and motor noise, the model's differential equations were solved iteratively by using the Euler 1-step integration (time step 1 ms). Noise was simulated as normally distributed random variable with a mean of 0 at each time step (see Equation 5), with equal noise factors and uncorrelated sensor and motor noise for the main modeling approach (see Discussion).

For fitting the noise level (determined by the noise factor) that corresponds to an optimal gain factor minimizing retinal image slip, which is equal to the model gain factor, 1,000 s of aVOR eye movement recordings at a frequency of 0.5 Hz and ±10° head motion amplitude was simulated with different sensor and motor noise factors. The 0.5 Hz stimulus condition was chosen because it is located well within the frequency bandwidth of the VOR. Determining the noise for the 1 Hz, ±5° positional amplitude yielded quantitatively similar results (not shown). It should be noted that for finding the noise level, the dynamic model of each animal is used, rather than a specific response.

For comparison of the eye movement variability, the noise level and the optimal gain factor were used to simulate an aVOR at four experimental conditions. The duration of each simulation was chosen to approximately match the duration of the experimental data sets at each frequency (0.1 Hz: 150 s, 0.2 Hz: 80 s, 0.5 Hz: 50 s, 1 Hz: 50 s). The simulated head and eye position data were then fed through the same data analysis procedure as the experimental data to generate comparable average results.

Simulated data were compared to experimental data by using the coefficient of determination *R*^2^ calculated as $R^{2}=1-SS_{res}/SS_{tot}\;$with $SS_{res}=\underset{i}{\sum }{\left(y_{i}-f_{i}\right)}^{2}$ being the residual sum of squares (data points *y*_*i*_, model points *f*_*i*_) and $SS_{tot}=\underset{i}{\sum }{\left(y_{i}-\bar{y}\right)}^{2}$ the total sum of squares ($\bar{y}$ is the mean of the data points). The coefficient of determination can become negative, if the model used is inadequate, that is, worse than just taking the mean of the data, and thus produces a residual sum of squares larger than the total sum of squares.

### Sequence of Analysis

**Figure 2:**
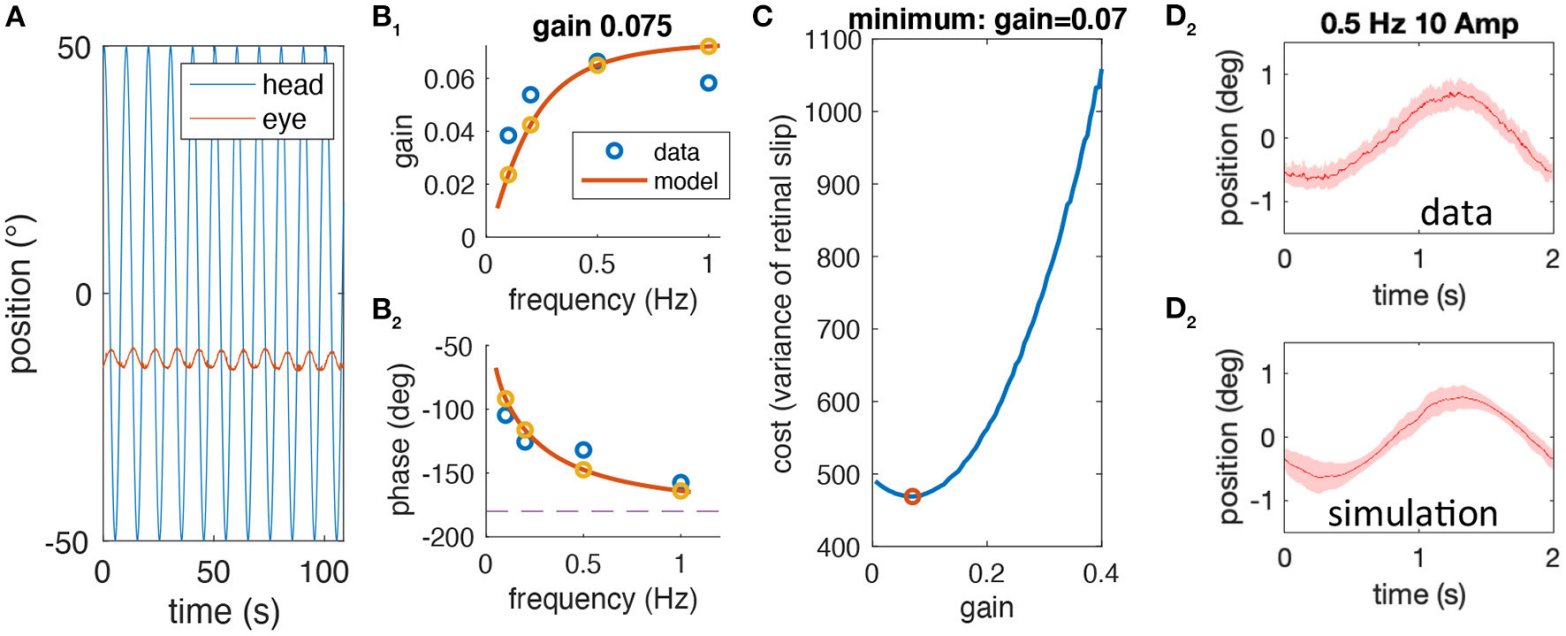
Sequential steps of the methodological approach. **(A)** Example of raw head (blue) and eye (red) motion data obtained from one animal at a stimulus frequency of 0.1 Hz. **(B)** gain **(B**_**1**_**)** and phase values **(B**_**2**_**)** derived from raw data (blue) for a single animal along with the model simulation of the best fitting model; the gain value for this animal is 0.075. **(C)** Simulated cost function (retinal image slip variance) plotted with respect to the gain factor using the model derived in **(B)** for different noise factors; here, the simulation is shown for the optimal gain factor that matches the animal's actual gain factor. **(D)** Averaged eye position response during rotation at 0.5 Hz for the data **(D**_**1**_**)** and the simulation **(D**_**2**_**)**; note that the average eye position trace of data and simulation (mean, red line) is similar due to the prior model fitting, but that the variability, which is also similar (shaded red) is a prediction based on the noise level and gain factor.

The following lists the complete sequence of steps performed to test whether the experimentally observed variability of aVOR eye position traces is compatible with our hypothesis, which proposes that low aVOR gain values optimize the retinal image slip. The different steps are shown in Figure 2 and explained below.

1) For each stimulus frequency, raw head and eye motion data (Figure 2A) are averaged over stimulus cycles to compute experimental gain and phase values (blue circles in Figures 2B1,2) for a given animal.
2) The computational model (see section Mathematical Modeling) is fit to the frequency response of each animal to extract individual model parameters (fitted model response, yellow circles and red lines in Figures 2B1,2). One of the model parameters is the aVOR gain factor for this animal.
3) The computational model is used to simulate responses at 0.5 Hz, ±10° amplitude with different noise factors, to calculate retinal image slip, and to find the noise level that requires an optimal gain factor corresponding to the experimentally observed VOR gain value (Figure 2C).
4) The predicted variability of eye position for the model simulation at the emerging noise level (Figure 2D1) is compared with the corresponding variability of the experimental data (Figure 2D2).

Note that the variability of the experimental data is an independent variable that has not been used in the entire process until the comparison in step 4. Thus, if our hypothesis is correct, i.e., that low aVOR gain values optimize retinal image slip, then the variability, predicted by the model, and the experimental variability should be quantitatively similar.

## Results

### Frequency Response

**Figure 3:**
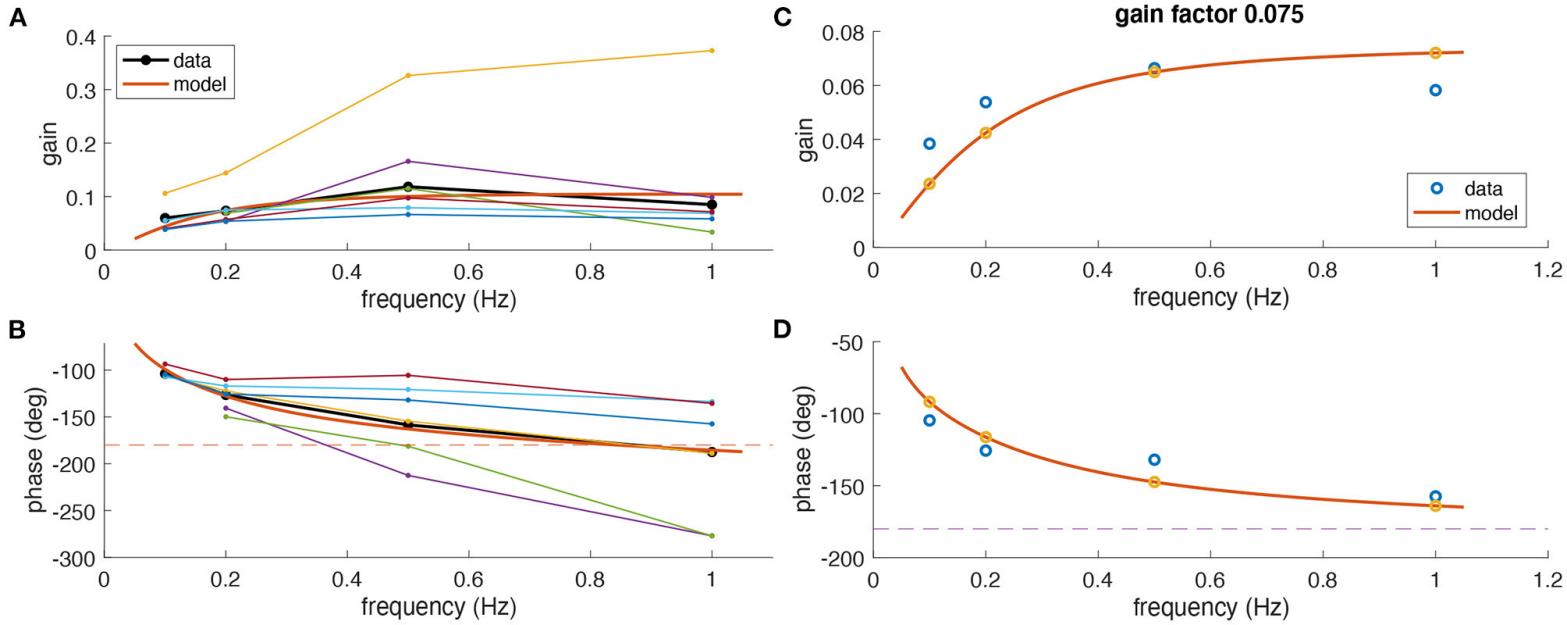
Comparison of experimental and modeling data. **(A,B)** Experimental gain **(A)** and phase values **(B)** for eye movements of *Xenopus* tadpoles (*n* = 6) (colored small dots) along with average gain and phase values (large black dots); the red curve depicts simulated gain and phase values obtained from the computational model (red line) of the tadpole aVOR that have been fitted to the average results (black); note that an eye movement with a phase shift of −180° [dashed line in **(B)**] and a gain of 1 would perfectly compensate for the head rotation at all frequencies. **(C,D)** Example experimental data (blue circles) obtained from one animal (same as in Figure 2) and modeling results (red curve, yellow circles), with gain and phase values plotted in **(C,D)**, respectively.

On average, experimentally observed gain values for all frequencies were rather low (<0.15) although with considerable differences between animals (Figure 3A). Based on the phase relation, responses were compensatory (−180°) on average only for rotations at 1 Hz but also differed strongly between animals (Figure 3B). As indicated above, a perfect aVOR response at a stimulus frequency of 1 Hz would require a phase shift of −180° but also a gain of 1. Experimentally, at frequencies of 0.5 and 1 Hz, we indeed found an optimal phase shift for some animals, but at the same time a gain far below unity. Thus, the low gain value is unlikely to derive from a compensation of an inappropriate phase shift, but must have other reasons, such as motor or sensory noise.

### Modeling of Stimulus Frequency-Dependent Responses

The computational model was fitted to the responses obtained at different frequencies to extract an individual model for each animal. As expected from the individually different response dynamics (Figure 3), the model parameters also reflected these differences and variations between animals. The overall coefficient of determination (*R*^2^, or variance explained by the model) between experimental and fitted phase values was 0.82, confirming a good model fit. The mean parameter values were for the integrator time constant 1.50 ± 1.73 s, the two muscle activation time constants 22 ± 17 and 21 ± 18 ms, and the model gain factor 0.14 ± 0.09.

### Determination of the Noise Level

**Figure 4:**
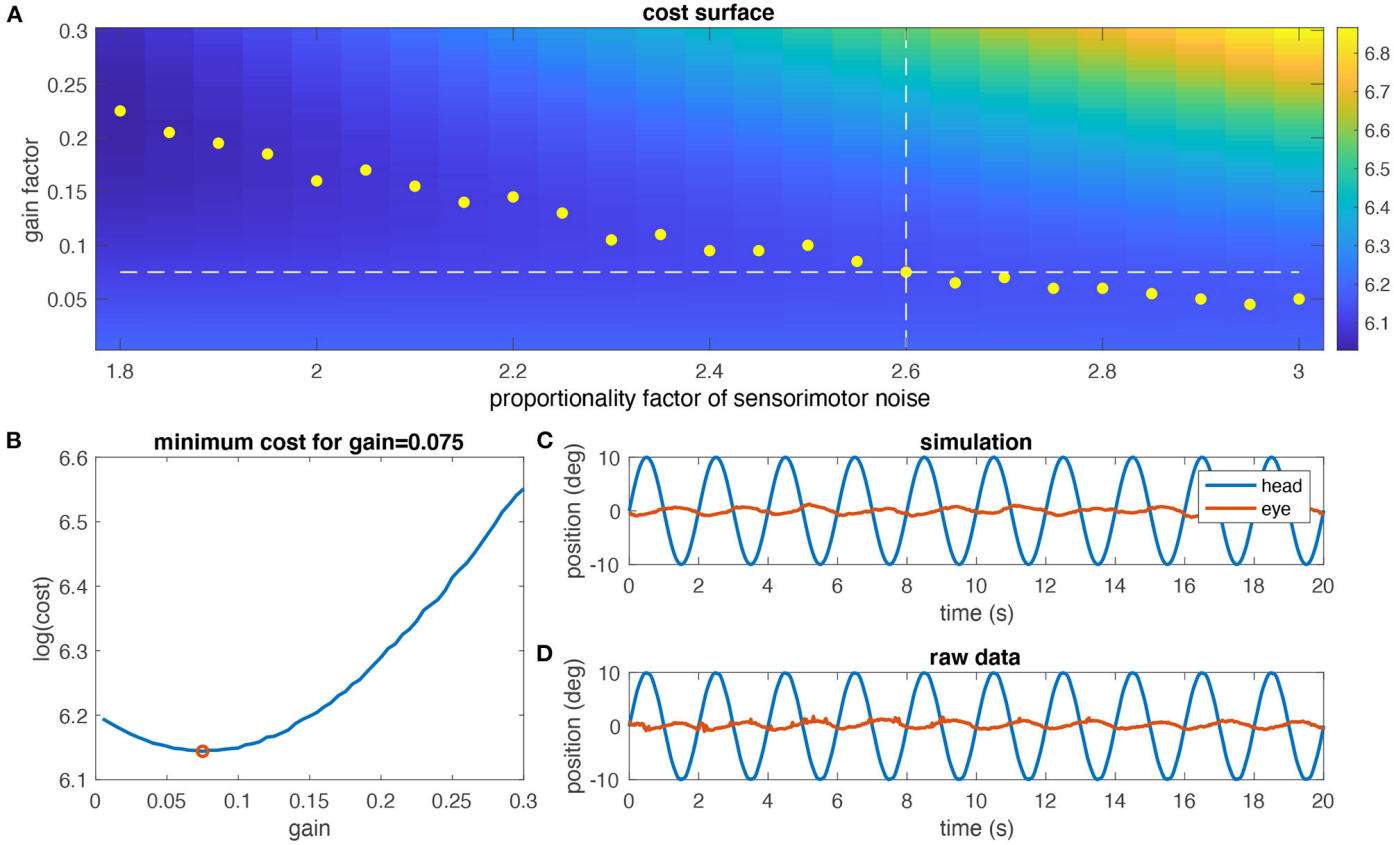
Determination of the noise level. **(A)** Simulated cost surface [as log (cost)] for one animal (same as in Figures 3C,D) and for eye movements at a stimulus frequency of 0.5 Hz and ±10° head motion amplitude plotted as function of the noise factor of sensorimotor noise and model gain factor; the cost of the aVOR has been quantified by calculating the variance of the retinal image slip; yellow dots indicate the minimum cost for each noise factor; the dashed horizontal line is the experimentally determined gain value, and the vertical dashed line the noise factor at which the optimal gain value is closest to the experimental gain value (2.6 in this case). **(B)** Section through the cost surface for a noise factor of 2.6; the optimal gain is 0.075, as shown in **(A)**. **(C)** Simulated eye movements (red) for the optimal gain value determined in **(A,B)**. **(D)** Raw eye movement recordings (red) for this animal for comparison.

Using the individual model derived above, aVOR responses to a stimulus frequency of 0.5 Hz and ±10° stimulus amplitude including sensory and motor noise were numerically simulated. For a given noise factor, a cost function for different gain values was computed by determining the variance of retinal image slip over one stimulus cycle. The optimal gain for the given noise factor was determined as the minimum of the cost function (yellow dots in Figure 4A). This computation was repeated for different noise factors to find the optimal gain that corresponds to the experimentally encountered aVOR gain at that particular frequency (white dashed lines in Figure 4A; red dot in Figure 4B). The same was performed for the alternative noise models.

### Comparison of Measured and Predicted Eye Movement Variability

**Figure 5:**
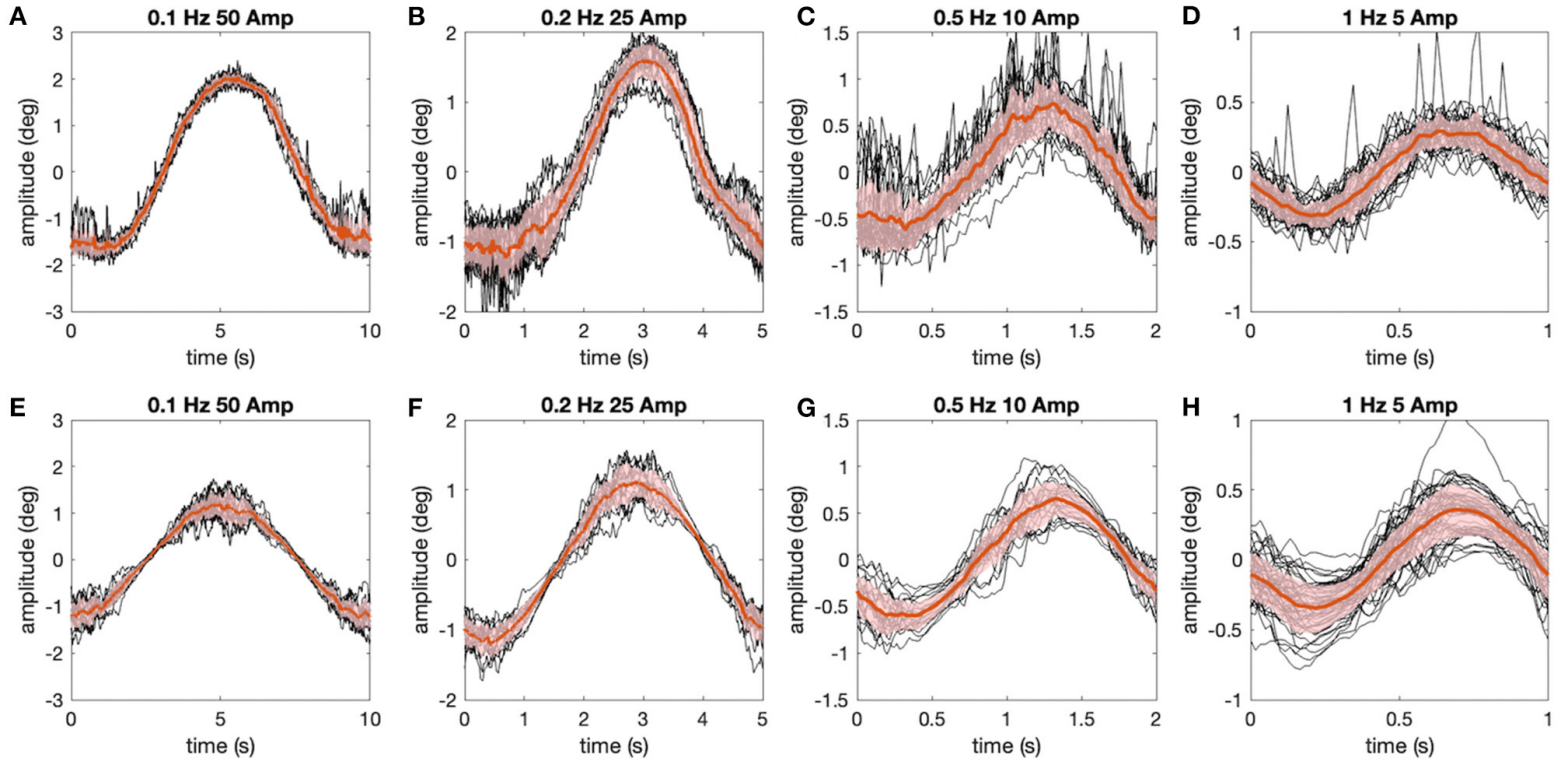
Comparison of experimentally measured and simulated eye movements. **(A–H)** Eye movements (raw traces in black, red lines show average) shown for all stimulus motion cycles at different stimulus frequencies depicted for the representative animal in Figure 4 **(A–D)**, along with corresponding simulations **(E–H)** of the data using the aVOR model (with appropriately adapted parameters) and optimal gain values to minimize the retinal image slip caused by signal-dependent sensor and motor noise (see also Figure 4); shaded areas indicate one standard deviation of the mean response.

Using the models obtained from individual animals and the noise factor determined above, all stimulus conditions were simulated (see Methods) and the simulated data (see Figure 4C) were analyzed using the same processing methods as applied to the real data (see Figure 4D). For the resulting average, simulated and real aVOR-related eye movements for each animal and frequency (see Figure 5 for an example), the eye movement variability was determined as average standard deviation of the eye position over one stimulus cycle (Figure 5, shaded regions). Importantly, the variability of the data has not been employed so far in the analysis or modeling process and thus represents an independent variable. The simulated variability derived from a prediction of the model under the assumption that the aVOR gain factor that has been determined from the data is optimized for the sensorimotor noise of an individual animal.

**Figure 6:**
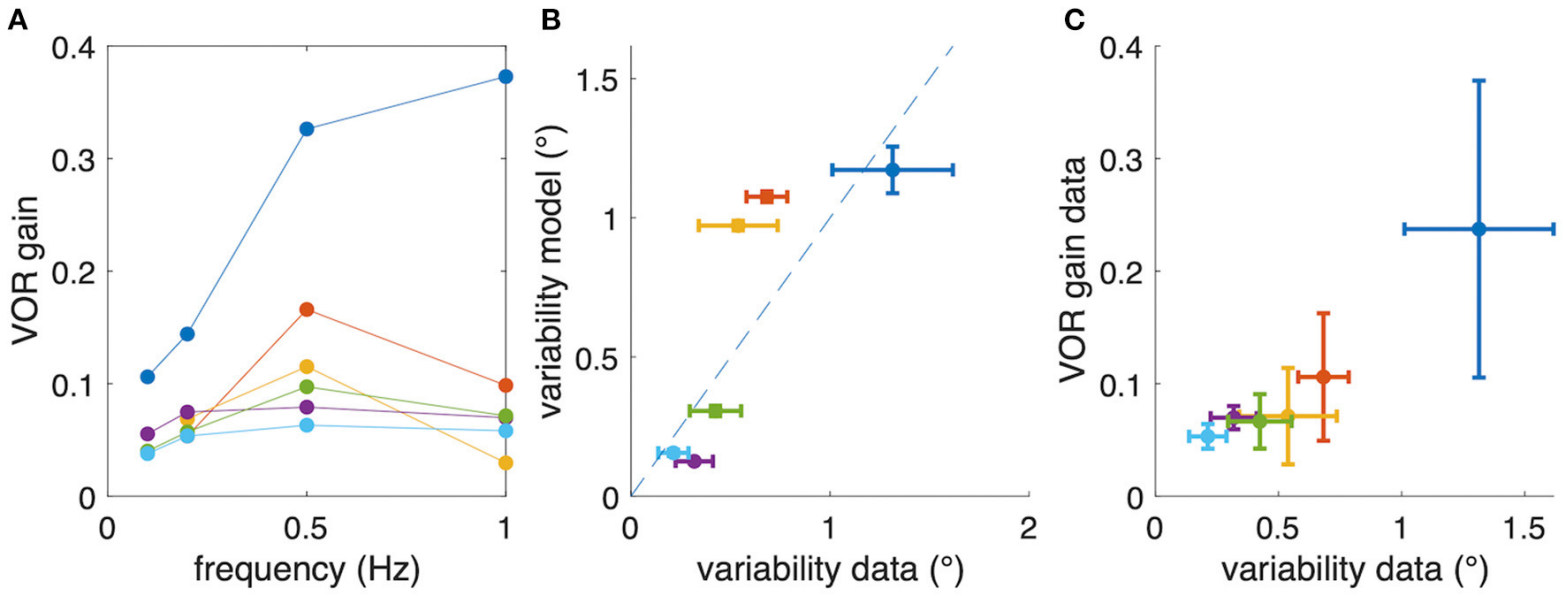
Relation between aVOR gain and variability of eye movements. **(A)** For comparison, aVOR gain values (see Figure 2) are shown for each animal separately in different colors. **(B)** Variability of eye movements for each animal plotted with respect to the predicted variability, i.e., the variability of the model simulations. **(C)** Variability of eye movements for each animal plotted with respect to the aVOR gain. Each data point in **(B,C)** represents the mean across four stimulus frequencies; error bars denote the standard deviation; note that for the animals depicted in yellow and orange only data for three frequencies were available.

Despite considerable inter-individual differences in aVOR gain factors (Figure 6A), the calculated variability, predicted by the model, corresponds to the experimentally determined variability (see Figure 6B, coefficient of determination *R*^2^ = 0.46, *n* = 6). A Bayes factor test corresponding to a paired *t*-test (15), comparing simulated (0.63 ± 0.49, mean ± SD) and experimental (0.58 ± 0.39) variability results in BF = 2.46, which is slightly in favor of the hypothesis that simulated and experimental variability are statistically not different. Thus, the similarity between predicted and experimentally occurring variability supports our hypothesis that small gain values, such as those found in *Xenopus* tadpoles, are possibly due to an optimization that minimizes retinal image slip. The alternative models show lower coefficients of determination (signal-dependent motor noise only: *R*^2^ = 0.104) or negative values (constant sensor noise only: *R*^2^ = −4.71; signal-dependent sensor noise only: *R*^2^ = −8.25), suggesting that these models are unable to adequately explain the data. However, also for those models, the resulting simulated eye movement variability was still in the same range as found experimentally.

**Figure 7:**
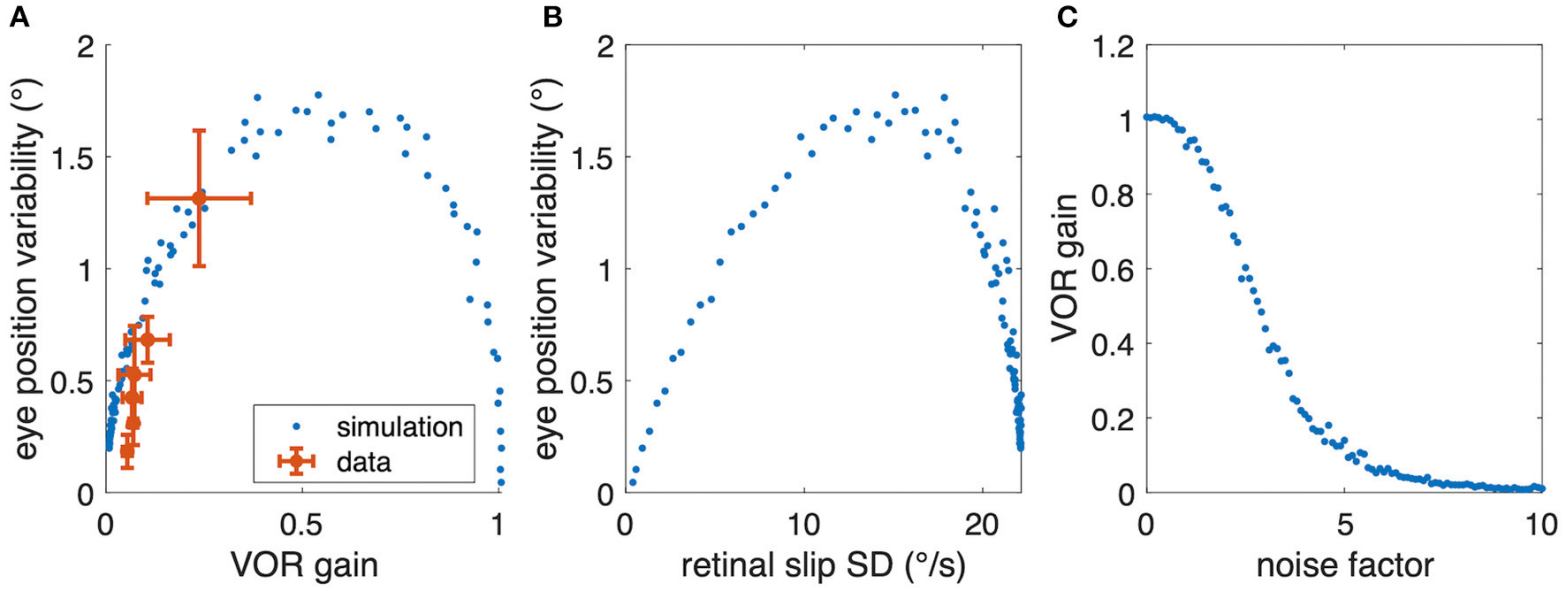
Relation between aVOR gain and variability of eye movements. **(A)** Replot of data from Figure 6C (red) together with model simulations of the standard aVOR model with varying sensorimotor noise and optimized aVOR gain (all other model parameters kept constant); the model predicts that for low aVOR gain values, the eye position variability will increase with increasing gain values (<0.5), but decrease again for higher values (>0.5). **(B)** Variability of eye movements for each animal plotted with respect to the retinal image slip [same simulations as in **(B)**]. **(C)** Optimal aVOR gain values [model simulations as in **(A,B)**] decreases with the sensorimotor noise factor.

However, when plotting the experimental aVOR gain factors with respect to the variability found in the actual data, smaller gain values are found to be related to smaller eye position variability (Figure 6C). This finding seems at a first glance to contradict our hypothesis, because intuitively one would expect that in the optimized condition a larger aVOR gain can only be possible if the eye movement variability becomes smaller. This intuition, however, is erroneous for the rather low VOR gain values of tadpoles. This can be easily demonstrated by simulating a sinusoidal aVOR with a standard set of model parameters and varying noise levels. Accordingly, model simulations predict that, for the small aVOR gain regime (gain values <0.5), eye position variability will increase with increasing gain (Figure 7A). For higher gain values (>0.5), the variability decreases again (Figure 7A). The reason for this particular outcome can be best understood when considering the extremes: if there is no noise, eye position variability will be zero, and the best possible aVOR gain (disregarding phase shifts) will be unity. This corresponds to the lower right corner of Figure 7A. If there is an extremely high noise level, the best option is to set the aVOR gain to zero such that the noise cannot control the eye movements. In this case, eye position variability will also be zero (with signal-dependent motor noise), because the eyes do not move. This situation corresponds to the lower left corner of Figure 7A. Between these two extremes, eye position variability will necessarily be larger than zero and, at some point, reach a maximum (close to a gain of 0.5). Retinal slip will be smallest for zero noise and unity gain, and highest for zero eye movement. Consequently, eye position variability and standard deviation of retinal image slip also show a non-linear relationship (Figure 7B), while optimal aVOR gain values, as expected, decrease with increasing sensorimotor noise (Figure 7C).

## Discussion

Our model simulations demonstrated that for minimizing the retinal image slip for successful gaze stabilization, both, the internal dynamics of the sensorimotor processing and the sensorimotor noise represent important parameters. The optimal aVOR gain factor that minimizes retinal image slip should be lower than unity for dynamics involving delays or phase shifts and for systems involving sensorimotor noise. To illustrate these theoretical considerations, a simple, linear dynamic model of the VOR with non-ideal parameters (small integrator time constant, uncompensated muscle activation) and signal-dependent noise at both the sensor and the motor level was simulated. The variance of retinal image slip across repeated trials was used as cost function to be minimized. To experimentally test our considerations, we analyzed measurements of the aVOR of *Xenopus* tadpoles, which showed particularly low aVOR gains. For these data, our hypothesis suggests that the low gains are associated with high internal sensorimotor noise. However, the level of internal sensorimotor noise cannot be simply inferred from eye movement measurements, because the experimentally measured variability is a consequence of the optimization, but does not directly reflect the internal noise from sensors or muscles. Therefore, we have chosen an approach to predict the variability of individual eye movements by using the aVOR model with parameters based on measured responses at different frequencies and by assuming that the individual aVOR gain minimizes retinal image slip according to internal dynamics and sensorimotor noise. The variability of the model simulations showed an excellent quantitative match with respect to the measured data, thus supporting our hypothesis.

### Optimality in Vestibular Processing

The present study is only one in a row of recent attempts to support the idea that vestibular signal processing underlying gaze stabilization or perception is optimized to account for the influence of internal noise (2, 16, 17). Vestibular processing during spatial orientation has long been described as optimal estimation [e.g., (18)], and more recent models also propose such an optimality strategy for the central processing of vestibular signals that assist in gaze stabilization [e.g., (19)]. Optimization has also been suggested as means to explain apparent deficits: perceptual vestibular thresholds of human subjects who fully recovered from unilateral vestibular deficits show a characteristic bilateral increase of the motion thresholds, which has been explained as being due to optimal adaptation to increased noise levels (2). More recently, it has been demonstrated that age-related changes in the dynamics of the human aVOR, that is, of the time constant, can be explained by an optimal estimation process that aims at adapting to the sensory signal-to-noise characteristics, which degrades with age (16). However, while all these studies consider optimal estimation in the presence of sensory noise, none of them considers motor noise together with the importance of retinal image slip as determining cost function.

### The Role of Dynamics Vs. Noise

While taking into account the internal dynamics of sensorimotor processing for our model, the deteriorating consequences of these dynamics (delays or phase shift) are not the main reason for the low gain values. A perfect compensatory aVOR, elicited at 1 Hz would require a unity gain and a phase shift of −180°. Experimentally, at 1 Hz an optimal phase shift on average (and very close to −180° for some individuals), but an average gain of 0.11, which is far below unity (Figure 2) was encountered. Thus, apart from semicircular canal size-related limitations in the sensitivity of the duct system in *Xenopus* tadpoles [(9, 10); see below], the generally low gain values in these animals cannot be simply explained by an inappropriate phase shift, but must have other reasons, such as motor or sensory noise as indicated above. Motor noise impacts motor commands at the level of motor unit recruitment (20) and would thus affect VOR responses independent of the respective sensory origin (otolith organs or semicircular canals). However, as shown previously, VOR gain values of responses activated by semicircular canal plus otolith organ stimulation are considerably higher than those of responses produced by semicircular canal stimulation alone: in *Xenopus* tadpoles at stage 54, the horizontal VOR at 1 Hz has been reported to express a gain of 0.26, while for the torsional VOR (semicircular canal plus otolith organs) the gain was ~0.6 (9, 10). Similar conclusions hold for combinations of vestibular and visual motion signals: in mice, the aVOR gain in light is twice as large as in darkness (6). Therefore, motor noise, even though it probably plays a considerable role, is likely insufficient to explain the low gain values of a horizontal, purely semicircular canal-driven aVOR. This thus requires reconsideration of the effect of sensory noise from the semicircular canals on aVOR responses and specifically on ocular motor responses related to retinal image slip.

### Alternative Explanations for Low VOR Gains

An alternative reason for the low VOR gain observed in *Xenopus* tadpoles could be that the extraocular motor system in these animals might simply be unable to generate faster eye movements. However, this is not the case given the occasional expression of saccadic eye movements with velocities that are considerably higher than required for a perfect VOR (21). Yet another possibility to explain low gain values in *Xenopus* tadpoles is that semicircular canal-evoked responses saturate much earlier than expected and thus cannot transmit head velocities >30°/s. However, data presented previously show that this explanation is also unlikely [e.g., (9)], and the approximately sinusoidal shape of aVOR responses provides no evidence for a potential saturation of the VOR at the applied stimulus intensities (21), supported by direct measurements of extraocular motoneuronal spike discharge at increasing stimulus amplitudes (22). While unusually low in darkness, the aVOR of *Xenopus* tadpoles in light is supplemented by efficient, visuo-motor reflexes that naturally assist the maintenance of gaze stability (21). Even though the overall gain of the aVOR in light is only mildly enhanced, the phase of the aVOR in light matches much better an expected compensatory response (21). In fact, visuo-motor responses such as the optokinetic reflex are particularly prominent at low-frequencies, although the natural frequency of swimming in tadpoles is even higher than the imposed head motion frequency applied in the current study (23). While the swimming frequency gradually decreases with developmental age, the tadpole head oscillates approximately sinusoidally at about 5 Hz with an amplitude of 20° at stage 53–54 (23). The rather stereotyped motion dynamics and profile is exploited by the gaze stabilizing circuitry in *Xenopus* tadpoles by recruiting spinal locomotor efference copies to directly drive compensatory eye movements during swimming (10, 24). Therefore, an alternative explanation for low VOR gain values might be that gaze stabilization in *Xenopus* tadpoles becomes relevant only during high-frequency active swimming but might be more dispensable during passive motion at lower frequencies. However, the contribution of locomotor efference copies to gaze stabilization during active swimming likely provides a means to avoid excessive sensory noise introduced by vestibular sensory signals, which is fully compatible with the outcome of the modeling approach in the current study. Thus, to answer the question whether low gain values are indeed due to excessive sensory noise, it will be necessary to directly measure the variability of afferent vestibular signals during head rotation.

### Limitations of the Current Study

The video capture of eye movements with a camera at 50 Hz likely introduces measurement noise that potentially contaminates the recordings and thus might critically influence the variability of the eye position data determined in the final step of the analysis. However, camera-induced measurement noise is, in contrast to our assumptions for internal noise, independent of the signal (at least at the low stimulus frequencies used here) and thus can be considered as forming an additive noise to eye position signals. In contrast, sensorimotor noise is signal-dependent and acts on head velocity and subsequent neural activity. In addition, even if the measured variability would be affected by the measurement noise, it would overestimate rather than underestimate the variability. This might in fact explain why, for 4 out of 6 animals, the experimental variability was higher than predicted by the model (Figure 6B). It should be noted that instead of comparing the variability of the eye velocity or retinal image slip we have chosen to compare eye position variability, because this avoids the need to calculate the temporal derivative of eye position data, which would increase the effect of high-frequency measurement noise.

A further limitation of the current approach is that the relation of sensor to motor noise in the processing of aVOR signals is at present unknown. In the seminal work by Harris and Wolpert (8) on saccadic eye movements, and also in later work on combined goal-directed eye-head movements (25), the motor noise was responsible for the variability in final gaze position. For the present work, there is strong evidence from vertical VOR performance [(9), see above] that motor noise alone cannot be responsible for low gain values. Similarly, when gaze stabilization is driven by locomotor efference copies during active swimming (10, 24, 26), the gain is ~0.6, demonstrating that higher VOR gain values are indeed possible, and that motor noise, which would affect all directional types of gaze stabilization components equally, cannot be the main reason for the observed low VOR gains. For human VOR responses it was recently reported that stimulus-dependent sensory noise is a likely explanation for the velocity-dependent variability of the VOR (16), matching the assumptions of the current study. However, for simplicity, in the present modeling approach equal amounts (in terms of noise factors) of sensor and motor noise were assumed, even though this assumption requires further exploration. Our finding that only sensor or only motor noise is unable to explain the data should be taken with caution until more data are available. It would be greatly beneficial to further investigate the dependency of variability on the eye position within the stimulus cycle, which can be clearly seen in the simulations (Figures 5E,F) and to a lesser extent also in the data (e.g., Figure 5B). One possibility for further experimentation would be to analyse the noise spectrum of eye movement responses, which, according to the model, differs for sensor noise (filtered by central processing and the eye plant) and motor noise (filtered only by the eye plant). Nonetheless, due to the presence and efficacy of the direct VOR pathway (27), these differences might only be subtle, and a direct measurement would be certainly more useful [see (28) for an exemplary study of vestibular afferents]. Therefore, future experiments would need to assess the level of sensor noise in vestibular nerve afferent responses vs. the level of motor noise, for example, when eye movements are driven by electrical stimulation at different levels of the processing (afferent semicircular canal nerve, vestibular nuclei, extraocular motor nuclei).

### Importance for Human VOR

The hypothesis of the current study may apply not only to other vertebrate species with low VOR gain values, but might represent a general principle applicable to all mechanistic types of gaze stabilization. For example, VOR in light, i.e., with vision, usually improves the gain considerably, which under the present hypothesis is easily explainable if vestibular sensory noise plays a significant role: the additional optokinetic visual input might decrease the overall sensory noise, indicating that the corresponding optimal gain can be increased. As emphasized above, healthy elderly subjects (1) or subjects with a history of vestibular damage (2) may show considerably lower VOR gain values [but see (3, 4) for preserved gains in elderly]. Again, this effect, observed in both groups of humans, could be explained by increased sensory noise; in addition, in elderly, increased motor noise might also play a role. Even for smooth pursuit eye movements the same considerations may apply: if retinal image slip is the most important cost, then lower pursuit gains, for example in elderly, may be related to higher sensorimotor noise. Hence, small VOR gain values in elderly and patients may be an optimal adaptation to the changed sensorimotor properties rather than derive from a sensory deficiency: without such adaptation, gaze stabilization would be worse. This would add to the growing evidence that observable changes in motor or sensory abilities in recovering patients or elderly subjects are better understood as a consequence of sensorimotor optimization strategies to partly compensate for the damage, rather than being a direct effect of the damage. However, a recent study (29) on VOR responses in control subjects and patients with unilateral vestibular damage found no relationship between VOR gain and VOR variability (measured as root-mean-square error of eye velocity relative to the mean response for the early part of head-impulse tests). This finding apparently contradicts our hypothesis for the human VOR. One possibility is that for the human VOR the present theory is not applicable, because in humans high gain might be more important in order to keep the fovea approximately on target instead of minimizing retinal image slip. It should be noted that keeping minimal foveal distance also predicts that with high signal-dependent sensorimotor noise levels, the optimal VOR gain should be lower than unity. Another possibility, supported by preliminary model simulations (not shown) and compatible with the present hypothesis, is that additional variability, introduced by the trial-to-trial variability of head velocity in head-impulse tests, has obscured the expected gain-variability relationship. Yet another recent study in humans (30) shows that changes in dynamics, and possibly also of the gain of the aVOR in patients with unilateral damage are a consequence of optimal adaptation to elevated sensory noise levels relative to the available signal. Nonetheless, more experimental studies, specifically in humans, are necessary to resolve the question whether low gain values for gaze stabilization may be an optimal adaptation to increased sensorimotor noise.

## Data Availability Statement

The raw data supporting the conclusions of this article will be made available by the authors, without undue reservation.

## Ethics Statement

The animal study was reviewed and approved by the Ethics Committee for animal experimentation of the Regierung von Oberbayern.

## Author Contributions

SG and HS conceived of the work, critically revised the work, and approved the final version. SG drafted the work, analyzed the data, and designed the model. All authors contributed to the article and approved the submitted version.

## Funding

This work was supported by Deutsche Forschungsgemeinschaft (STR 478/3-1 and GL 342/2-1) and the Bernstein Center for Computational Neuroscience Munich (BT-1).

## Conflict of Interest

The authors declare that the research was conducted in the absence of any commercial or financial relationships that could be construed as a potential conflict of interest.

## Publisher's Note

All claims expressed in this article are solely those of the authors and do not necessarily represent those of their affiliated organizations, or those of the publisher, the editors and the reviewers. Any product that may be evaluated in this article, or claim that may be made by its manufacturer, is not guaranteed or endorsed by the publisher.
